# Text Messaging to Improve Hypertension Medication Adherence in African Americans: BPMED Intervention Development and Study Protocol

**DOI:** 10.2196/resprot.4040

**Published:** 2015-01-02

**Authors:** Lorraine R Buis, Nancy T Artinian, Loren Schwiebert, Hossein Yarandi, Phillip D Levy

**Affiliations:** ^1^University of MichiganDepartment of Family MedicineAnn Arbor, MIUnited States; ^2^Wayne State UniversityCollege of NursingDetroit, MIUnited States; ^3^Wayne State UniversityDepartment of Computer ScienceDetroit, MIUnited States; ^4^Wayne State UniversityDepartment of Emergency Medicine and Cardiovascular Research InstituteDetroit, MIUnited States

**Keywords:** mobile phone, text messaging, hypertension, blood pressure, African Americans, medication adherence, mobile health

## Abstract

**Background:**

Hypertension (HTN) is a major public health concern in the United States, with almost 78 million Americans age 20 years and over suffering from the condition. Moreover, HTN is a key risk factor for health disease and stroke. African Americans disproportionately shoulder the burdens of HTN, with greater prevalence, disease severity, earlier onset, and more HTN-related complications than age-matched whites. Medication adherence for the treatment of HTN is poor, with estimates indicating that only about half of hypertensive patients are adherent to prescribed medication regimens. Although no single intervention for improving medication adherence has emerged as superior to others, text message medication reminders have the potential to help improve medication adherence in African Americans with uncontrolled HTN as mobile phone adoption is very high in this population.

**Objective:**

The purpose of this two-phased study was to develop (Phase I) and test in a randomized controlled trial (RCT) (Phase II) a text message system, BPMED, to improve the quality of medication management through increasing medication adherence in African Americans with uncontrolled HTN.

**Methods:**

In Phase I, we recruited 16 target end-users from a primary care clinic, to assist in the development of BPMED through participating in one of three focus groups. Focus groups sought to gain patient perspectives on HTN, medication adherence, mobile phone use, and the use of text messaging to support medication adherence. Potential intervention designs were presented to participants, and feedback on the designs was solicited. In Phase II, we conducted two pilot RCTs to determine the feasibility, acceptability, and preliminary efficacy of BPMED in primary care and emergency department settings. Both pilot studies recruited approximately 60 participants, who were randomized equally between usual care and the BPMED intervention.

**Results:**

Although data collection is now complete, data analysis from the two pilot RCTs is still ongoing and results are expected in 2015.

**Conclusions:**

This study was designed to determine preliminary feasibility and acceptability of our approach among African Americans with uncontrolled HTN in primary care and emergency department settings. Results from these studies are of great interest as little work has been done to document the use of text message medication reminders to improve HTN-related outcomes, particularly within underserved urban minorities.

**Trial Registration:**

Clinicaltrials.gov NCT01465217; https://clinicaltrials.gov/ct2/show/NCT01465217 (Archived by WebCite at http://www.webcitation.org/6V0tto0lZ).

## Introduction

### Background

Almost 78 million Americans age 20 years and over suffer from hypertension (HTN) [1], which is a key risk factor for health disease and stroke (the first and fourth leading causes of death in the United States, respectively) [2]. HTN is more prevalent among non-Hispanic blacks (42.0%) than non-Hispanic whites (28.8%) [3], a pattern that has persisted for 50 years [4,5]. Moreover, African Americans shoulder burdens of greater disease severity, with earlier onset and more HTN-related complications than age-matched whites [6].

Medication adherence for the treatment of HTN is poor, with estimates indicating that only about half of hypertensive patients are adherent to prescribed medication regimens [7,8]. To date, no single intervention for improving medication adherence has emerged as superior to others; however, those that include reminders have been shown to have positive effects on adherence and patient outcomes [9].

Mobile phones and text messages have become widely integrated into routine daily life and may offer a simple and non-labor intensive strategy for enhancing medication adherence. Work by Lawton et al suggests that the use of innovative approaches to medication management, such as text message interventions, may be useful at increasing medication adherence in adults [10]. Furthermore, the US Department of Health and Human Services recently published an environmental scan of the state of the science of text messaging to improve consumer health knowledge, behaviors, and outcomes, which reports that text messaging has been shown to increase treatment compliance, including medication adherence [11].

While African American adults use the Internet less frequently than whites (80% vs 87%) and have less access to home broadband (62% vs 74%), there are no significant differences in adoption of cellphones (92% and 90%) or smartphones (56% and 53%) [12], suggesting that mobile health (mHealth) is a viable strategy to reduce HTN-related disparities. Moreover, text messaging is the most common activity performed on a mobile phone among American adults, with 81% of mobile phone owners reporting that they text message [13].

### Study Objective

The purpose of this two-phased study was to develop (Phase I) and test (Phase II) a text message system to improve the quality of medication management through increasing medication adherence in African Americans with uncontrolled HTN. Phase I was accomplished through user-centered design principles incorporating direct feedback from target end-users, and Phase II will be accomplished through the completion of two pilot randomized controlled trials (RCT) with patients recruited from primary care and emergency department (ED) settings (target of approximately 60 participants each). The RCT design of our evaluation provides the opportunity to potentially determine if use of the intervention, BPMED, has an effect on primary and secondary outcome measures, compared to usual care controls, at 1-month follow-up.

### Hypotheses

In our Phase II primary care pilot RCT, we hypothesize that individuals assigned to the text message intervention will have (1) a greater increase in medication adherence from baseline to 1-month follow-up as compared to individuals receiving usual care treatment, (2) a greater increase in medication adherence self-efficacy from baseline to 1-month follow-up as compared to individuals receiving usual care treatment, and (3) a greater reduction in systolic and diastolic blood pressure (SBP and DBP) from baseline to 1-month follow-up as compared to individuals receiving usual care treatment.

Because it is expected that not all participants in the ED pilot RCT will be undergoing active treatment for HTN at the time of enrollment, Hypotheses #1 and #2 will not be as salient in the ED trial due to lack of meaningful baseline adherence and medication self-efficacy measures.

## Methods

### Overview

The goal of this study was to develop and test an automated text message system to increase medication adherence among African Americans with uncontrolled HTN (using BPMED) and was designed in two parts. In Phase I, we recruited target end-users to participate in focus groups to gather target end-user feedback to be used in the design of the BPMED intervention. In Phase II, we initially sought to test the feasibility, acceptability, and preliminary efficacy of BPMED in a pilot RCT of uncontrolled hypertensive African Americans recruited from a primary care setting. The final protocol closely mirrored what was originally proposed to the funding agency (see Multimedia Appendix 1 for original review summary statements). We subsequently received funding to conduct a parallel study to test the feasibility, acceptability, and preliminary efficacy of BPMED in a pilot RCT of a similar population recruited from an ED setting. Whittaker et al previously proposed a multi-step process for the development and evaluation of mHealth interventions, which included the following stages: conceptualizing, formative research, pretesting, pilot study, RCT, and qualitative follow-up [14]. In the development and evaluation of our own BPMED intervention, we utilized many of the same processes endorsed by Whittaker et al, and in the following sections, we delineate how our own process followed much of their already established process.

### Intervention Development

#### Target End-User Recruitment

We approached the development of BPMED with user-centered design principles in mind. To gain user perspective on the design of BPMED, we conducted formative research, one of the several steps in mHealth intervention development and evaluation outlined by Whittaker et al [14], through focus groups that sought to gain patient perspectives on HTN, medication adherence, mobile phone use, and the use of text messaging to support medication adherence. We recruited target end-users to participate in one of three focus groups. These participants were recruited through targeted recruitment letters sent to primary care patients who met clinical eligibility requirements as identified in a retrospective chart review of electronic medical records of primary care patients at our recruitment site. This method was an efficient means of recruiting focus group participants, as it greatly accelerated participant recruitment and ensured that we were more likely to reach potential participants who were likely to meet our eligibility criteria. To be eligible for inclusion in our Phase I focus groups, participants had to meet the following eligibility criteria: self-identified African American, age ≥18 years, diagnosis of HTN based on International Classification of Diseases (ICD-9) codes in the medical record, had uncontrolled HTN on two successive clinic visits prior to screening (clinic SBP >140 mm Hg, DBP >90 mm Hg or SBP >130, DBP >80 for those with diabetes or kidney disease) as documented in the medical record, taking at least one antihypertensive medication, own a mobile phone capable of receiving and sending text messages, ability to pay for and obtain HTN medications, and English speaking.

#### Focus Groups

Focus groups were conducted by the Wayne State University (WSU) Center for Urban Studies (CUS) and independent staff who were not directly involved in the study itself. Although study staff were present to introduce themselves, thank participants for their participation, and to explain the purpose of the study, all study staff excused themselves from the room prior to the start of the focus groups, and all focus group facilitation and moderation was conducted by trained WSU CUS staff members. To control for any potential effect that the race of our focus group moderators had on the outcome of the focus groups, the CUS staff members conducting the focus groups were African American females. At the outset, instead of written informed consent, all participants were given an information sheet that explained the purpose of the study, participation expectations, risks, benefits, and compensation. Prior to the focus groups, all participants were provided a meal and were asked to fill out a survey assessing demographics, medication adherence (using the Morisky Medication Adherence Scale; MMAS) [15], and mobile phone use.

After the completion of the survey, all focus groups were conducted using the same structured focus group script that was broken down into three separate parts. In the first part, facilitators explained the purpose of the study and all procedures for the focus groups, including participant expectations, focus group guidelines, and study compensation. In the second part, focus group members discussed HTN, medication regimens, medication adherence, and reasons for medication non-adherence. Finally, focus group participants were asked about mobile phones and text message use, and feedback was solicited regarding participant perceptions of using text messages for three different potential intervention functions: (1) to enable participant self-reporting of medication adherence, (2) to provide medication reminders, and (3) to receive educational information pertaining to HTN. In addition, participants were asked to identify what additional features would be useful in a text messaging program to increase medication adherence, as well as to identify potential problems that may be encountered by using text messaging for this purpose. The focus group protocol included prompts to solicit participant feedback on messaging frequency and content. Each of the three focus groups lasted approximately 90 minutes. At the conclusion of the focus groups, participants were given a US $25 cash incentive. In total, 16 individuals participated in one of three focus groups (n=6, n=4, and n=6, respectively). All methods were approved by the Wayne State University Institutional Review Board (#0410810B3E).

#### Data Analysis

During the focus groups, a CUS staff member served as the focus group moderator, while a second CUS staff member served as a dedicated note taker, capturing detailed notes on participant responses, and audio recording the focus groups for later review. After the focus groups, the two CUS staff members reviewed the audio recordings and supplemented the detailed notes with direct quotations and any opinions expressed by focus group participants that were not captured in the original notes. The CUS staff members also analyzed the notes and recordings for themes pertaining to HTN and medication adherence, as well as mobile phone and text message use. CUS staff also noted whenever consensus or majority opinions were expressed by focus group members related to potential BPMED functionality. Finally, all suggestions for possible BPMED functionality were prioritized by study staff according to participant majority consensus and ultimate feasibility of implementation. All survey data collected prior to the focus groups were analyzed with descriptive statistics in STATA 11.0.

#### Focus Group Sample

Focus group participants were primarily female (87.5%, 14/16) and ranged in age from 34-67 years (mean 50.8, SD 9.6). All participants had at least a general education development (GED) or high school diploma. Despite the level of education of our sample, annual combined household incomes for the previous year were quite low with 31.3% (5/16) earning less than US $10,000, and 25.0% (4/16) earning between $10,000 and $19,999. In terms of mobile phone use, 100.0% (16/16) of focus group participants reported that they carried their mobile phone all day, every day, and had the ability to send and receive text messages. Regarding mobile phone plans, 66.7% (10/15) of participants reported that they had their mobile phone plans for 1 year or more, and 62.5% (10/16) report that unlimited text messaging was included in their mobile phone plan. Only 6.3% (1/16) report that they had used a prepaid mobile phone. Although text messaging capabilities were pervasive, only 56.3% (9/16) reported that they used text messaging daily. See Table 1 for a complete breakdown of focus group participant characteristics.

The complexity of antihypertensive medication regimens varied within our sample as 56.3% (9/16) of participants reported taking one antihypertensive medication, 12.5% (2/16) taking two medications, and 31.3% (5/16) reported taking three or more different medications. Based on MMAS scores, the majority of our sample were considered to have low medication adherence (62.5%, 10/16), while 18.8% (3/16) were medium and 18.8% (3/16) were high adherers.

#### Focus Group Findings

Based on the themes that emerged from our three focus groups, it was clear that participants did not always take their medications as prescribed, with participants stating: “I have to think about it: did I take that medicine? I’m afraid I’ll take a double dose, so I say ‘well, forget it’ ” and “I try to take my medication every day…The dosing is really easy, but I might take it later, like two or three hours later”.

Reasons for not taking medications as prescribed varied, but simply forgetting to take their meds was cited by the majority of focus group participants as the primary reason for medication non-adherence. In addition, participants also mentioned issues related to fears of doubling up, stopping medications when HTN symptoms subsided, and undesirable side effects as reasons for non-adherence. Participants reported developing their own tricks for remembering to take medications, such as “Every time Steve Harvey [is on], I know it’s time to take my blood pressure medicine”. Participants overwhelmingly supported the use of text messaging, citing beliefs such as: “That’s the way people talk now” and “In this day and age, everybody texts…[You are going to] automatically look, just to see who it is”.

In addition, the vast majority of participants thought that text message reminders would be helpful in improving medication adherence. Specifically, participants requested the ability to customize the frequency and timing of medication reminders each day. In addition, focus group participants indicated a preference for receiving educational text messages with information on topics such as nutrition and HTN symptoms, although a few expressed concerns about costs associated with receiving additional text messages. Although there was great support for receiving text message reminders for medication adherence, participants were adamant that they did not want to use text messaging for self-monitoring their adherence back to the study team. As our initial conceptualization of our intervention was based on the Theory of Self-Regulation and relied heavily on participant self-monitoring, this was key information to inform the development of BPMED.

**Table 1:** Focus group participant characteristics (n=16, except where indicated).

Characteristic		n (%)
Age, mean (SD)		50.8 (9.6)
**Gender**		
	Female	14 (87.5)
	Male	2 (12.5)
**Highest level of education** ^a^		
	High school diploma or GED	8 (50.0)
	Some college	4 (25.0)
	Bachelors degree	3 (18.8)
	Graduate degree	1 (6.3)
**Marital status** ^a^		
	Single, never married	6 (37.5)
	Married	1 (6.3)
	Divorced	7 (43.8)
	Widowed	2 (12.5)
**Annual household income, US$**		
	<10,000	5 (31.3)
	10,000-19,999	4 (25.0)
	20,000-39,999	3 (18.8)
	40,000-59,999	2 (12.5)
	≥60,000	2 (12.5)
**Employment status** ^a^		
	Part time	4 (25.0)
	Full time	3 (18.8)
	Retired	2 (12.5)
	On disability	3 (18.8)
	Laid off / unemployed	4 (25.0)
**Mobile phone plan is prepaid (requires phone cards)** ^a^		
	Yes	1 (6.3)
	No	15 (93.8)
**Length of current mobile phone plan ownership** ^b^		
	<1 month	0 (0.0)
	1-3 months	2 (13.3)
	4-6 months	1 (6.7)
	7-12 months	2 (13.3)
	>1 year	10 (66.7)
**Who pays for mobile phone** ^c^		
	Self	10 (76.9)
	Spouse	0 (0.0)
	Family member other than spouse	1 (7.7)
	Friend	2 (15.4)
**Frequency of text message use** ^a^		
	Never	3 (18.8)
	A few times per month	3 (18.8)
	A few times per week	1 (6.3)
	Daily	9 (56.3)
**Does mobile phone plan include text messaging** ^a^		
	Yes, unlimited text messaging is included in plan	10 (62.5)
	Yes, a limited number of text messages are included in mobile phone plan	3 (18.8)
	No	2 (12.5)
	Don’t know	1 (6.3)

^a^Sum total does not equal 100% due to rounding error.

^b^n=15.

^c^n=13.

### BPMED Intervention Design

Based on focus group participant feedback, we developed our 1-month intervention to provide automated text message medication reminders that would be sent to participants at the time of their choosing. Because we did not have access to all participant therapeutic regimens as documented in their medical records, we had to rely on participant self-reports of dosing regimens. Moreover, as dosing times vary between participants, sending daily reminders at times that participants were not taking their medications was considered not useful. As such, we allowed participants to set the number of text message reminders they wished to receive each day, as well as timing of the messages, to suit their needs.

In addition to medication reminders, eight educational messages (two/week) were developed and added to the intervention. These educational messages were based on HTN management recommendations from the American Heart Association and were focused on topics of smoking cessation, the importance of limiting dietary sodium, coping with stress, nutrition, weight reduction, limiting alcohol use, and physical activity. A weekly satisfaction question asking participants to rate their satisfaction with the BPMED intervention was also added. Finally, there were several study-related text messages that were used to enroll participants into BPMED, set up medication reminders, and remind participants to schedule their 1-month follow-up data collection appointment (see Table 2 for a complete listing of all text messages included in BPMED). All text messages were pretested by content experts with experience working in our target community. Pretesting, another step endorsed by Whittaker et al in mHealth intervention design and evaluation [14], helped to ensure that our messages were accurate, clear, and appropriately tailored to our target population.

BPMED utilized a double opt-in architecture. First, study staff enrolled each participant in an online participant management system with their name, phone number, and participant identification. As a part of this enrollment process, participants were required to read a statement that further clarified expectations for involvement in the study, as well as the number of text messages participants could expect to send/receive as a part of their participation. Directions for how to opt-out of the text message intervention were also provided. Once participants read the statement and checked a box indicating that they agreed, participants received an automated text message asking to confirm the participants’ desire to enroll in the text message intervention. After agreeing a second time to use the text message intervention, participants were fully enrolled and were instructed on how to set medication reminders.

Although we had initially intended to ground our work with text messaging in a Theory of Self-Regulation framework, as previously mentioned, formative research revealed that target users were adamantly opposed to using text messaging to self-monitor their medication adherence. With our shift toward providing simple text message medication reminders, BPMED became more closely aligned with a Health Belief Model framework with medication reminders serving as cues to action, and educational messaging serving to increase participant perceived benefits of behavior change and self-efficacy, as well as reduce perceived barriers to behavior change.

**Table 2:** Text messages included in BPMED by type.

Type of message	Content of message	Day of intervention that message was sent
Medication reminder	BPMED: Hello [first name], this is your [reminder time] reminder to take your medication. Please don’t forget!	Sent daily at times specified by participant
Educational	BPMED: The DASH diet works! Eat foods rich in whole grains, fruits, vegetables, and low-fat dairy, while limiting saturated fat and cholesterol.	3
	BPMED: Physical activity lowers blood pressure. Aim for 30 minutes each day over small chunks or all at once. Try a 30 min brisk walk or three 10 min walks!	6
	BPMED: Smoking can increase blood pressure. If you are a smoker and want help quitting, call 1-800-QUITNOW or talk to your doctor.	10
	BPMED: Foods high in sodium (salt) can increase blood pressure. Try to limit your sodium intake to 1500 mg/day, including what is in and what is added to food.	13
	BPMED: Did you know that 30 minutes of moderate physical activity can help make your blood pressure medications work more effectively and help you feel better?	17
	BPMED: To manage your blood pressure, limit your alcohol consumption to no more than two drinks per day for men and no more than one drink per day for women.	20
	BPMED: To help reduce sodium intake, use spices instead of salt in cooking and at the table. Flavor foods with herbs, citrus, vinegar, or salt-free blends.	24
	BPMED: Did you know that blood pressure rises as body weight increases? Losing even 10 pounds can lower blood pressure and your risk of chronic disease.	27
Satisfaction assessment	BPMED: We would like your feedback [first name]. From 1-10 (with 10 being best), how satisfied are you with the program? Reply BPMED RESPONSE [number of intervention week] and your rating.	7, 14, 21, 28
Study-related	BPMED: You've chosen to receive text message medication reminders. If you wish to proceed text BPMED AGREE FIRSTNAME LASTNAME to 37717. Standard message rates apply.	0 (baseline)
	BPMED: [first name], thanks for participating in this study. To begin, please text BPMED START FIRSTNAME LASTNAME to 37717.	0 (baseline)
	BPMED: Thanks for participating! Once you set up your reminders, you will start receiving them tomorrow and will continue to get them for one month.	0 (baseline)
	BPMED: To set up reminders, text BPMED TIME and the times you want to get texts, ie BPMED TIME 9am 5am 730pm, to 37717. Please mind the spacing.	0 (baseline)
	BPMED: Thanks [first name]. You’ve chosen to receive reminders at [reminder time 1] [reminder time 2]…	Whenever participant requests a new reminder
	BPMED: Hello [first name]. If you have not yet set up your one month follow-up appointment, please give us a call at 313-577-4107 to do so.	26
	BPMED: Thank you for your study participation. Today is your last day of reminders.	30

### Phase II Pilot Randomized Controlled Trials

#### Trial Design

**Figure 1:**
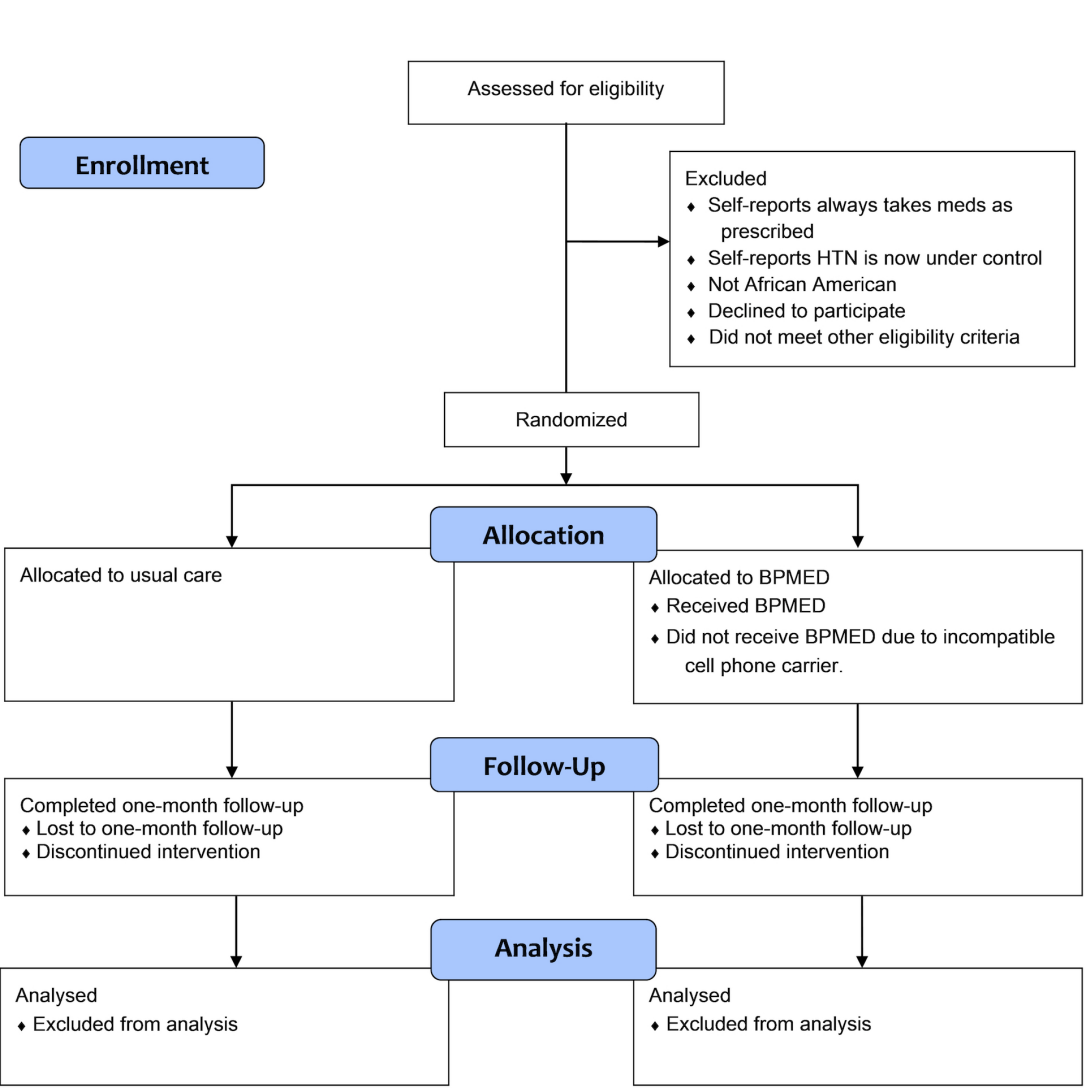
Participant flow through BPMED trials.

In Phase II, we sought to conduct two 1-month pilot RCTs with patients recruited from primary care and ED settings. These trials sought to enroll approximately 60 participants each, who were randomized equally to receive either usual care or the BPMED intervention (see Figure 1 for participant flow through the trials). We chose these specific settings to reflect the way patients in our community engage with the health care system and to assess potential scalability of our intervention to real world clinical practice. Although Whittaker et al advocates the use of non-randomized pilot tests in the development and evaluation of mHealth interventions [14], we chose to use an RCT design of BPMED compared to usual care control so that we may be able to determine potential intervention effects on primary and secondary outcome measures at 1-month follow-up.

#### Human Subjects Protections

The methods utilized in the two pilot RCTs were approved by the Wayne State University Institutional Review Board (#0410810B3E).

#### Participant Recruitment

To enlist participants for the primary care pilot RCT, a combination of recruitment methods were used including targeted recruitment letters mailed directly to potentially eligible participants who met clinical eligibility requirements as identified through retrospective records analysis of electronic medical records, recruitment posters displayed in primary care clinic exam rooms, and direct provider referral. Interested, potentially eligible participants were screened via phone, and those who met inclusion criteria were scheduled for a baseline data collection visit. Potential participants who previously participated in the Phase I focus groups were not excluded from participation in the pilot RCTs. To recruit participants for the ED pilot RCT, patients meeting clinical eligibility criteria were identified initially through real-time monitoring of the ED tracking board by trained research assistants. Recruitment, screening, and enrollment were conducted onsite in the Detroit Receiving Hospital ED and typically occurred directly after patient discharge from the ED.

#### Inclusion/Exclusion Criteria

To be eligible to participate, participants were required to self-identify as African American, be at least 18 years of age, have a diagnosis of HTN based on ICD-9 codes documented in the medical record, own a mobile phone capable of receiving and sending text messages, and be English speaking. Additional inclusion criteria specific to the primary care pilot included having uncontrolled HTN on two successive clinic visits prior to screening (clinic SBP >140 mm Hg, DBP >90 mm Hg or SBP >130, DBP >80 for those with diabetes or kidney disease) as documented in the medical record, and be taking at least one antihypertensive medication. Additional inclusion criteria specific to the ED pilot included having elevated blood pressure on successive measurements. Exclusion criteria included reporting strict adherence to medication regimens, receiving hemodialysis, plans to move >50 miles from the recruitment site within the next 3 months, diagnosis of resistant HTN documented in the medical record, plans to terminate mobile phone contract during the next month, compliance risk, and/or other major health problems that would make participation in this study difficult.

#### Intervention

All trial participants were fully informed about the risks and benefits of participating in this study, and written informed consent was obtained from all participants. Prior to randomization, all participants completed a baseline survey assessing demographics, mobile phone use, medication adherence, and medication adherence self-efficacy, as well as having their blood pressure taken. Primary care patients had been previously instructed to bring all antihypertensive medications with them to the baseline data collection visit so that pill counts could be taken to be used as an additional measure of medication adherence. In the ED trial, given that many participants were not currently taking medications to treat their HTN, all ED trial participants were given a 35-day supply of an appropriate antihypertensive medication. After completing all baseline measures, participants were block-randomized with a 1:1 ratio to either the usual care control or BPMED intervention group. Intervention group participants were then enrolled in BPMED and trained on how to use the program. All participants, regardless of trial assignment, were asked to come back for a 1-month follow-up visit where medication adherence, medication adherence self-efficacy, and blood pressure measures were assessed. In addition, intervention group participants completed a brief BPMED satisfaction questionnaire and open-ended interview at 1-month follow-up. This qualitative follow-up among intervention group participants post trial is advocated by Whittaker et al in the development and evaluation of mHealth interventions [14]. All participants received US $25 at the conclusion of baseline and 1-month follow-up data collection visits, for a possible total of US $50/participant. Any participant who indicated that text messaging was not included in their mobile phone plan was reimbursed US $0.20/text message sent or received as a part of their participation in this study. Finally, parking expenses were covered for primary care trial participants.

#### Outcomes

Medication adherence was the primary outcome of interest in this study and was measured using the MMAS [15], pill counts, and an additional self-report of medication adherence. For the primary care trial, medication adherence change from baseline to 1-month follow-up was of particular interest. In the ED pilot, adherence at follow-up was the primary outcome of interest due to the fact that some participants were not taking an antihypertensive medication at baseline. Secondary outcome measures of interest included medication adherence self-efficacy as measured by the Medication Adherence Self-Efficacy Scale [16], blood pressure, and participant satisfaction with BPMED. We also conducted post-trial qualitative interviews and questionnaires to assess BPMED participant perceptions of the program. In addition to participant outcomes, logs of unintended system down time will be analyzed to understand whether participant reminders were sent as expected.

#### Trial Statistical Methods

Descriptive statistics will be used to describe participant characteristics and intervention satisfaction at follow-up. Categorical data will be displayed as frequency and percentages, and where appropriate, chi-square tests will be used for comparison. Continuous variables such as medication adherence, medication adherence self-efficacy, and blood pressure will be expressed as mean (standard deviation), and means will be compared using two-tailed unpaired independent samples *t* tests. Regression models, controlling for variables such as gender, age, and other potentially important confounders, will be used to predict improvements in primary and secondary outcomes. All statistical analysis will be carried out using STATA version 12.0.

## Results

Data collection from both pilots is now complete, and results from these pilots are expected to be published in early 2015.

## Discussion

### Principal Findings

This paper outlines the methodology of a two-phased study designed to develop (Phase I) and test (Phase II) the feasibility, acceptability, and preliminary efficacy of text message medication reminders to improve medication adherence in African Americans with uncontrolled HTN. Through the completion of the two pilot RCTs, we seek to add to the growing evidence base of mobile phone–based mHealth approaches for chronic disease self-management. With a rigorous study design, we hope to advance the field of mHealth beyond single group quasi-experimental research designs. Moreover, we hope to demonstrate the feasibility and acceptability of our approach among an urban African American population that suffers from considerable HTN-related health disparities and is often overlooked as a good candidate for technology-based behavior change interventions.

### Limitations

Perhaps the largest limitation to this study is our reliance on a short-term 1-month follow-up. This 1-month follow-up period was chosen for several reasons. First of all, this study was designed as a pilot project and was primarily concerned with showing feasibility and acceptability of our approach, with secondary emphasis on demonstrating preliminary efficacy. In addition, our 1-month follow-up period was chosen out of an initial concern of the stability of mobile phone numbers in this population.

Another limitation to our approach is our dependence on small sample sizes. To address this, depending on the differences in results of our two pilots, we will consider pooling the results if appropriate. We also acknowledge a lack of health care provider feedback in the design of BPMED and that many members of the care team, including physicians, nurses, and pharmacists, may have valuable insight to provide in the development of this sort of intervention. Given that this is a consumer-facing intervention that could be used independent of an established patient-provider relationship, we made the conscious decision to focus exclusively on the patient perspective in our design, but we do understand that additional provider feedback from a variety of care team members may have improved our ultimate design.

Finally, the lack of additional rigorous objective measures of medication adherence, such as the use of electronic pill bottles or other objective devices, may weaken our primary outcome measure. The decision not to use such devices was made out of budgetary necessity. Future work should seek to use more rigorous measures of medication adherence.

### Comparison With Prior Work

The evidence supporting long-term efficacy of text message medication reminders is largely missing from the literature [11], and the evidence supporting short-term efficacy has been mixed. Improvements in medication adherence as a result of receiving text message reminders have been demonstrated for adherence to antiretroviral therapy (ART) for people living with HIV/AIDs [17], as well unspecified medication regimens [18], and a variety of conditions and treatments, including type 2 diabetes [19], glaucoma [20], schizophrenia [21], and asthma [22]. However, other studies focused on text message reminders for acne medications [23] and oral contraceptives [24] have found no effect. A recent systematic review of text message reminders for health services by Kannisto et al [25] identified 60 studies that met inclusion criteria. Of those, 63% were focused on studies that utilized text message reminders for medication or treatment, whereas the other 37% were focused on clinical appointment reminders. Kannisto et al report that 77% of studies report improved outcomes on primary outcome measures of interest. Despite largely positive data suggesting the efficacy of the use of text message reminders for health care services, the authors acknowledge that more well-conducted studies are still needed to build the evidence base for this approach [25].

Although several studies have investigated the use of text message medication reminders for a variety of diseases, conditions, and therapeutic regimens, little work has been done to study this approach within the context of HTN. Moreover, few studies have been published to date that focus on the use of this approach within an urban, African American population. This trial protocol marks one of the first RCTs testing the efficacy of text message medication reminders on medication adherence among hypertensive African American participants.

### Conclusions

This study was designed to determine preliminary feasibility and acceptability of our approach in a cohort of African Americans with uncontrolled HTN in primary care and ED settings. Through our 1-month pilot RCTs, we also expect to demonstrate preliminary efficacy of BPMED to provide support for further exploration of the use of text message medication reminders to improve HTN-related outcomes in underserved urban minorities.

## Multimedia Appendix 1

Initial summary statements from AHRQ.

## Abbreviations

ART: antiretroviral therapy
CUS: Center for Urban Studies
DBP: diastolic blood pressure
ED: emergency department
HTN: hypertension
ICD-9: International Classification of Diseases version 9
mHealth: mobile health
MMAS: Morisky Medication Adherence Scale
RCT: randomized controlled trial
SBP: systolic blood pressure
WSU: Wayne State University
