# Supplementary material for: Text Messaging to Improve Hypertension Medication Adherence in African Americans: BPMED Intervention Development and Study Protocol
Source: JMIR Res Protoc. 2015 Jan 2;4(1):e1. doi: 10.2196/resprot.4040 (PMC4296103; doi:10.2196/resprot.4040)
Supplement: Supplementary file 1 [file resprot_v3i4e82_app1.pdf]

SUMMARY STATEMENT
(Privileged Communication)

Release Date: 03/29/2010

PROGRAM CONTACT:
Angela Lavanderos
(301) 427-1505
anunley@ahrq.gov

Application Number: 1 R21 HS019092-01

Principal Investigator

BUIS, LORRAINE R PHD

Applicant Organization: WAYNE STATE UNIVERSITY

Review Group: HQER
Health Care Quality and Effectiveness Research

Meeting Date: 03/03/2010
Council: MAY 2010
Requested Start: 07/01/2010

RFA/PA: PAR08-269
PCC: CP3

Project Title: Text Messaging to Improve Hypertension Medication Adherence in African Americans
SRG Action: Priority Score: 161 Percentile: 1.8
Human Subjects: 30-Human subjects involved - Certified, no SRG concerns
Animal Subjects: 10-No live vertebrate animals involved for competing appl.
Gender: 1A-Both genders, scientifically acceptable
Minority: 1A-Minorities and non-minorities, scientifically acceptable
Clinical Research - not NIH-defined Phase III Trial

Project Year	Direct Costs Requested	Estimated Total Cost
1	113,334	172,268
2	82,218	124,971
TOTAL	195,552	297,239

ADMINISTRATIVE BUDGET NOTE: The budget shown is the requested budget and has not been adjusted to reflect any recommendations made by reviewers. If an award is planned, the costs will be calculated by Agency grants management staff based on the recommendations outlined below in the COMMITTEE BUDGET RECOMMENDATIONS section.

RESUME/SUMMARY OF DISCUSSION: This R21 grant application entitled “Text Messaging to Improve Hypertension Medication Adherence in African Americans “ is in response to the PA08-269 Exploratory and Developmental Grant to Improve Health Care Quality through Health Information Technology (IT) (R21) from Wayne State University, MI; Dr Lorraine R. Buis is the Principal Investigator (PI). The proposed study is to improve quality of medication management, safety and clinical outcomes for high-risk population. The study will be conducted in two phases In Phase 1 data will be obtained from focus groups to create the text messages and identify factors that will enhance usability, in Phase 2 the text messaging intervention will be pilot-tested for one month. There are strengths in the application. The PI is well qualified to conduct this research project and a well-recognized leader in the area of mass media and human computer interactions. The study of successful management of hypertension and poor adherence to prescribed BP medications has highly significance, because uncontrolled high blood pressure is one of a major public health concern and leading cause of cardiovascular disease, stroke and renal failure among African Americans, effective data collection with adequate privacy and security protection has the potential to use existing technology to increase the likelihood of acceptability and usability in this population. The approach to develop and pilot test base on cell phone text massaging and receiving feedback to increase medication adherence among African Americans with uncontrolled hypertension is a novel approach. The study is strong and promising preliminary data is presented that document the feasibility of the proposed method. However, few weaknesses that need to be addressed appropriately were noted in this proposed study. The interdependence of Phases II with the Phase I outcomes is a concern, especially because several design elements cannot be determined as timing and number of medications for targeted population. There are not enough details about blinding of data collectors, and the study may be insufficiently powered due to the poor description of the sample size estimation procedure and the plan for analyzing the data. Overall, this is the straight forward promising study regarding the utilization of Health Information Technology to manage the patient’s safety and effectiveness of service, the reviewers recommend this application for further consideration with appropriate level of enthusiasm” Excellent” range.

DESCRIPTION (provided by applicant): Uncontrolled high blood pressure (HBP) is a major public health concern and leading cause of cardiovascular disease worldwide. The HBP crisis is particularly onerous to African Americans as they are disproportionately more susceptible to HBP than non-Hispanic White Americans. Poor adherence to prescribed medication regimens is a major problem, as only about half of patients who have been diagnosed with hypertension adhere to prescribed medications. Mobile phones and text messages are becoming widely integrated into routine daily life and may offer a simple and non-labor intensive strategy for improving the quality of medication management through enhancing medication adherence. This proposed research will be conducted in two distinct phases addressing three specific aims. For Specific Aim 1 (Phase I), we propose to conduct focus groups with participants from our target population in order to obtain feedback to guide the development of a mobile phone text message system that seeks to improve adherence to antihypertensive medications. For Specific Aim 2 (Phase II), we propose to pilot test the newly developed text message intervention with a randomized controlled trial. For Specific Aim 3 (Phase II), we propose to ascertain participant perceptions of intervention effectiveness and satisfaction in order to guide further system refinement. In Phase I, African Americans with uncontrolled hypertension (n=24-32) will be recruited to take part in one of four focus groups that will help guide the development of the text message intervention. In Phase II, African Americans with uncontrolled hypertension will be randomized to receive usual care (n=30) vs. the text message intervention (n=30). The primary outcome in this pilot will be change in medication adherence at one month follow-up; secondary outcomes include change in medication self-efficacy and systolic and diastolic blood pressure at one month follow-up, as well as participant satisfaction with the text message intervention. The proposed research will utilize the Wayne State University (WSU) Center for Urban Studies to conduct high quality and professionally run focus groups, the WSU Division of Computing and Information Technology's Broadcast Message Service infrastructure for the delivery of text messages, the WSU Center for Health Research for statistical analysis and grant management assistance, and a diverse study team from a variety of disciplinary backgrounds at WSU. As a result, the proposed research leverages the

considerable local resources to investigate an innovative and much needed intervention for this high-risk population.

PUBLIC HEALTH RELEVANCE: Poor adherence to prescribed medication regimens is a major problem, particularly among African Americans with uncontrolled high blood pressure. Mobile phones and text messages may offer a simple and non-labor intensive strategy for enhancing medication adherence. Due to its reliance on technologies already adopted by individuals, this application seeks to develop and test an innovative, adaptable, and highly scalable text message intervention for improving medication adherence in our target population.

CRITIQUE NOTE: The sections that follow are the essentially unedited, verbatim comments of the individual committee members assigned to review this application. The attached commentaries may not necessarily reflect the position of the reviewers at the close of group discussion, nor the final majority opinion of the group. The above RESUME/SUMMARY OF DISCUSSION represents the evaluation of the application by the entire committee."

CRITIQUE 1

Significance: Does this study address one of the three health IT research areas of interest to AHRQ? Does the application focus on a gap in the literature or the field that needs to be addressed? Does the application adequately recognize and describe the limitations in the generalizability of the results of this project and any planned subsequent work and demonstrate the applicability of these research findings to other settings and circumstances?

The purpose of this 2-year R21 application is to develop and pilot test an automated text message intervention to increase medication adherence among African Americans with uncontrolled hypertension. In phase I of the study, focus groups will be conducted to obtain information from the targeted population to guide the development of the text messaging intervention delivered by a mobile phone device. Phase II will consist of pilot testing the newly developed test message in a RCT to determine if there is an increase in medication adherence to antihypertensive medications 1-month following the intervention. In addition, perceptions of effectiveness and satisfaction with the intervention will be examined. The primary outcome variable is change in medication adherence; secondary outcomes include medication self efficacy, blood pressure measurements, and patient satisfaction.

Strengths: It is a well-written investigation that plans to use existing technology (cell phone text messaging) as an interventional modality to improve medication adherence for uncontrolled hypertension. Inasmuch as HTN is a precursor to major cardiovascular disorders, interventions that can maximize its control and minimize its sequelae, are significant and timely. Moreover, the use of existing technology that is already incorporated into everyday life increases the likelihood of acceptability and usability in this population, as well as in other populations, settings, and circumstances. In addition to potential transferability, it may be a cost effective means to increase adherence and self-management while decreasing disparate care. The application is congruent with Health IT goal to enhance health decision making.

Approach:

Strengths: The specific aims are clear and match the overall purpose of the study. The Theory of Self-Regulation (self-monitoring, goal setting, and feedback) is used as the conceptual framework for intervention development and study implementation. The study will be conducted in two phases. In Phase I, end-user data will be obtained from focus groups (n = 24-32) to create the text messages and identify factors that will enhance usability. In Phase II, the text messaging intervention will be pilot-tested for one month in a second sample of participants (n = 60) with 30 individuals receiving usual

care and 30 individuals receiving usual care plus the text messaging intervention. Other extraneous variables that can influence medication adherence (comorbidities, stress, sleep, depression) will be examined.

Weaknesses: Because Phase II is dependent on the outcome of Phase I, several design elements cannot be determined; e.g. timing and number of medications for text message reminders.

There is some concern about the size of the sample and the accuracy of the power analysis to detect differences in the variables of interest. Additionally, given the sample size and the intent of the study, it may be premature to pose hypotheses. The study may not be sufficiently powered to adequately test the hypotheses.

The investigators may want to explore other variables that could influence the results such as length of time diagnosed with HTN.

Innovation:

Strengths: The use of cell phones as a health technology modality is innovative and transferable across populations.

Investigators: Are the PD/PI and other key personnel appropriately trained and well suited to carry out this work? Does the PD/PI and investigative team bring complementary and integrated expertise to the project (if applicable)? Is there adequate PD/PI support allocated throughout the research period or a well-justified reallocation of leadership during the course of the research project when PD/PI's support is less than suggested twenty-percent level? Does the application demonstrate the investigators' aptitude to identify and address weaknesses found either in the application or encountered during the conduct of the research project? Does the application demonstrate that the project team will have adequate administrative structure and processes in place to oversee the successful conduction of the proposed study?

Strengths: Primary investigator is well suited to lead this research project, and academically prepared in the area of mass media and human computer interactions. As an early investigator, Dr Buis has assembled a research team that has experience in medical adherence, hypertensive populations, computer science and statistics to support achievement of the study aims. Their previous investigators support and provide background for the proposed project.

Environment:

Strengths: The institutional, clinical, and technology resources at Wayne State University are sufficient to accomplish the study aims. The investigators have a demonstrated track record in enrolling minorities as research participants.

Privacy and Security Protections in the Development and Implementation of Health IT System: Has the applicant adequately described the privacy and security policies and features of the health IT system being developed, implemented, or used in the proposed study? Has the application adequately described the process for identifying and addressing privacy and security issues related to the exchange of sensitive health information and encouraging the involvement of patients to the extent appropriate? Has the application adequately considered patients' access to audit records, disclosure to patients regarding collection and use of electronic data, patient notification of security breaches, patient consent process, and ability for patients to decline participation in electronic data collection? Comments (Required):

Strengths: Privacy and security protections are adequate.

Weaknesses: No concerns.

Protection of Human Subjects from Research Risks:

Strengths: The human subject's protection plan is acceptable.

Inclusion of Women and Minority Subjects: Acceptable. It is estimated that 65% of the sample will be female. African Americans are the target population.

Inclusion of AHRQ Priority Populations: Elderly, rural, inner city, low income, chronic care, disabled

Weaknesses: The PI does not specifically address AHRQ priority populations.

Budget and Period of Support:

Strengths: The budget is acceptable.

Summary of Strengths and Weakness:

Strengths:

1. The use of readily accepted technology (cell phones) for delivery of a health care intervention (text messaging)
2. Conceptually guided and well articulated plan for accomplishing the study aims.

3. Experienced research team.

Weaknesses:

1. Some design ambiguity but understandable.
2. The study may be insufficiently powered.

CRITIQUE 2

Significance:

Strengths: This is short-term preparatory and feasibility study of an automated text message system to improve the quality of medication management by assisting individuals in self-monitoring their medications. Thus, it is addressing one of the main health IT research areas of interest to AHRQ. The study will address major gaps in the field by including: a theoretical basis for the design, the target population African Americans located in Detroit with a large AA population, and will be one of the first to focus on hypertension. The limitations of the study relative to sample size and recruitment challenges are recognized. Investigators anticipate that through the development of this text messaging system it will be possible to test different types of messages with different populations and health problems/behaviors.

Approach:

Strengths: The PI and co-I mentor have both successfully completed studies addressing adherence (to physical activity and medication) with the use of technologies (telehealth and web-based). Two proposed intervention components including text message logging and automated feedback regarding medication adherence goals both influence the cycle of self-regulation demonstrating a sound theoretical base. The entire first phase of the project is devoted to system development utilizing user-

centered design principles. Four focus groups will be held with members of the target population to obtain their input on multiple intervention components including structure of the flow, strategies for successful implementation etc. Following the completion of the feasibility study future input will be obtained from the target population related to their satisfaction. A particular strength of the measures is the attention to multiple measures of adherence to medications. Further, there is a sound fidelity plan in place. The aims, theoretical framework, and analyses are well integrated. The investigators do a good job of acknowledging the potential problem of phones being disconnected during the course of the study and have plans to minimize the problem.

Weaknesses: Blinding of data collectors was not addressed which is of minor concern.

Innovation:

Strengths: Using of text messaging both as a means of logging medication adherence and receiving feedback is a highly novel approach. It has potential benefit for a number of health behaviors beyond medication adherence. Using cell phone technology is a novel way to intervene with a low income at risk population. The investigators make a very sound case for capitalizing on the high use of cell phones and text messaging in this population.

Investigators:

Strengths: The PI and key personnel are appropriately trained and well suited to carry out this work. The PI is a new investigator with expertise in information technology. and has publications in both the use of technology and diverse populations. Dr. Artinian is ideally suited as a co-PI mentor. and brings experience in conducting clinical trials with the same population and she is an expert in adherence to hypertension medications. Two computer science experts and a statistician bring complementary and integrated expertise to the proposed project. These key members at approximately 5% across the course of the two years (with exception of statistician) should be adequate to provide the needed consultation to this new investigator.

Environment:

Strengths: Letters of support are in place for the two proposed data collection sites. The proposed study will benefit from collaboration with these study sites as well as from the *WSU Division of Computing & Information Technology* which will provide the infrastructure to assist in developing the proposed text messaging intervention.

Privacy and Security Protections in the Development and Implementation of Health IT System:

Strengths: Acceptable: all electronic data will be accessible only with a login and protected password; system access to electronic data will be limited to authorized individuals. The WSU WebMail uses industry standard SSL encryption.

Protection of Human Subjects from Research Risks: Acceptable

Inclusion of Women and Minority Subjects: Acceptable: Women, children (18) and minorities (all African American)

Inclusion of AHRQ Priority Populations: Low income, urban

Budget and Period of Support: No concerns.

Summary of Strengths and Weakness:

Strengths: This is a highly innovative intervention that is addressing a high priority area- medication adherence in a vulnerable population. The intervention is guided throughout by a theoretical model. Most importantly the target population is involved in the intervention development from the inception through completion of the feasibility study. The investigative team brings together expertise from information technology, nursing science (adherence) and computer science. If successful this technology should be easily transferable to other behaviors for which adherence can be a problem.

Weaknesses: Blinding of data collectors should be addressed.

CRITIQUE 3

Significance:

Strengths: The investigators propose to use cell phone text messaging to increase patient compliance with regard to administration of antihypertensive medication. A positive result would lead to a reduction in cardiovascular disease severity, less hospitalization, reduced morbidity and mortality.

Approach: Are the conceptual, design, methods, and analytical plan adequately developed, well-integrated, well-reasoned, and appropriate to the specific aims of the project? If proposed, how appropriate are the process and clinical outcome measures to be used? To what extent are patients and/or families directly involved in the design, development and/or selection of the health IT application or is adequate justification of why their involvement is not feasible or appropriate provided? To the extent that staff, patients and families are directly involved in the health IT implementation, is their perspective on the use and/or utility of the health IT sought or an adequate justification of why such information is not feasible or appropriate provided?

Strengths: The proposed investigation will allow simple, but effective data collection.

Innovation: Very innovative.

Investigators: The investigators are qualified to conduct the investigation.

Environment:

Strengths: There is a high proportion of AHRQ targeted populations in the patient base. Therefore, it is likely that a substantial number of African American subjects with poorly controlled hypertension will be recruited.

Privacy and Security Protections in the Development and Implementation of Health IT System:

Strengths: Data security and patient confidentiality are well addressed.

Protection of Human Subjects from Research Risks: Acceptable.

Inclusion of Women and Minority Subjects: Acceptable.

Inclusion of AHRQ Priority Populations: Acceptable.

Budget and Period of Support: The proposed budget is reasonable and overhead is 52%.

Summary of Strengths and Weakness: Very important topic with a strong likelihood of positive outcome.

CRITIQUE 4

The sections of the application that provided the rationale for the choice of sample size and the analysis may not have been reviewed carefully by the project statistician as they lacked detail that is generally expected in these sections. The description of the sample size estimation procedure and the plan for analyzing the data are distinct weakness of the application.

THE FOLLOWING RESUME SECTIONS WERE PREPARED BY THE SCIENTIFIC REVIEW OFFICER TO SUMMARIZE THE OUTCOME OF DISCUSSIONS OF THE REVIEW COMMITTEE ON THE FOLLOWING ISSUES:

PROTECTION OF HUMAN SUBJECTS: ACCEPTABLE

INCLUSION OF WOMEN PLAN: ACCEPTABLE

INCLUSION OF MINORITIES PLAN: ACCEPTABLE

INCLUSION OF AHRQ PRIORITY POPULATIONS: ACCEPTABLE. Elderly population, rural areas, disables, inner cities, low income and minority subjects also will be included in this study.

COMMITTEE BUDGET RECOMMENDATIONS: The budget was recommended as requested.

MEETING ROSTER

Health Care Quality and Effectiveness Research
Health Services Research Initial Review Group
AGENCY FOR HEALTHCARE RESEARCH AND QUALITY
HQER 1
March 03, 2010 - March 04, 2010

CHAIRPERSON

BALLARD, DAVID J., MD, MSPH, PHD
SENIOR VICE PRESIDENT AND CHIEF QUALITY OFFICER
BAYLOR HEALTH CARE SYSTEM
EXECUTIVE DIRECTOR & BHCS ENDOWED CHAIR
INSTITUTE FOR HEALTH CARE RESEARCH AND IMPROVEMENT
DALLAS, TX 75206

MEMBERS

AHLERS, DONNA CLAIRE, RN *
WELLNESS CONSULTANT
HEALTHY HORIZONS
WINTERVILLE, NC 28590

BAKER, DAVID W., MD *
PROFESSOR
FEINBERG SCHOOL OF MEDICINE
NORTHWESTERN UNIVERSITY
CHICAGO, IL 60611

CAREY, TIMOTHY S, MD, MPH *
PROFESSOR OF MEDICINE
SHEPS CENTER FOR HEALTH SERVICES RESEARCH
UNIVERSITY OF NORTH CAROLINA AT CHAPEL HILL
CHAPEL HILL, NC 275997590

DARDEN, PAUL M II, MD
PROFESSOR OF PEDIATRICS
HEAD OF GENERAL AND COMMUNITY PEDIATRICS
OUHSC DEPARTMENT OF PEDIATRICS
OKLAHOMA CITY, OK 73104

DELONG, ELIZABETH R., MD, PHD *
PROFESSOR & DIRECTOR
DEPT OF BIOSTATISTICS & BIOINFORMATICS
CO DIRECTOR, OUTCOMES RESEARCH &
ASSESSMENT GROUP HOCK PLAZA
DURHAM, NC 27705

DOWNS, JOHN B, MD *
PROFESSOR
UNIVERSITY OF FLORIDA
COLLEGE OF MEDICINE
DEPT. OF ANESTHESIOLOGY
DUNNELLON, FL 34432

ESCOBAR, GABRIEL J, MD
PHYSICIAN & RESEARCH SCIENTIST
KAISER PERMANENTE
DIVISION OF RESEARCH
OAKLAND, CA 94612

HERRINTON, LISA J, PHD
RESEARCH SCIENTIST III
KAISER PERMANENTE
DIVISION OF RESEARCH
OAKLAND, CA 94612

JACOBSON, ROBERT M, MD
PROFESSOR OF PEDIATRICS
MAYO CLINIC
ROCHESTER, MN 559050001

KANE, ROBERT L., MD
PROFESSOR
UNIVERSITY OF MINNESOTA
SCHOOL OF PUBLIC HEALTH
MINNEAPOLIS, MN 55455

KROTH, PHILIP J, MD
ASSISTANT PROFESSOR
HEALTH SCIENCES LIBRARY AND INFORMATICS CENTER
UNIVERSITY OF NEW MEXICO
ALBUQUERQUE, NM 87131

KULASINGAM, SHALINI L, PHD *
ASSISTANT PROFESSOR
UNIVERSITY OF MINNESOTA
MINNEAPOLIS, MN 55454

MEREDITH, LISA SEIDEL, PHD
SENIOR BEHAVIORAL SCIENTIST
RAND CORPORATION
SANTA MONICA, CA 904072138

PALACIOS, CESAR , MD, MPH *
EXECUTIVE DIRECTOR
PROYECTO SALUD CLINIC
WHEATON, MD 20902

PETITTI, DIANA B, MD *
PROFESSOR OF BIOMEDICAL INFORMATICS
ARIZONA STATE UNIVERSITY
PHOENIX, AZ 85004

RAHMAN, MAHBOOB , MD
ASSOCIATE PROFESSOR
CASE WESTERN RESERVE UNIVERSITY
CLEVELAND, OH 441064982

RANDALL, LESLIE L, BSN, RN
EPIDEMIOLOGIST
LAPWAI, ID 83540

ROWAN, PAUL J, PHD *
ASSISTANT PROFESSOR
SCHOOL OF PUBLIC HEALTH
DIVISION OF MANAGEMENT, POLICY
AND COMMUNITY HEALTH
UNIVERSITY OF TEXAS-HOUSTON
HOUSTON, TX 77030

SCOTT, LINDA D, BSN, MSN, PHD
PROFESSOR & ASSOCIATE DEAN FOR GRADUATE PROGRAMS
KIRKHOF COLLEGE OF NURSING
GRAND VALLEY STATE UNIVERISTY
GRAND RAPIDS, MI 49503

SILVERSTEIN, MARC D., FACP, MD
CHIEF DECISION SCIENTIST
INSTITUTE FOR CLINICAL ECONOMIC REVIEWS
MASSACHUSETTS GENERAL HOSPITAL
BOSTON, MA 02114

SIMMONS, SANDRA FAYE, PHD *
ASSOCIATE PROFESSOR
VANDERBILT UNIVERSITY
CENTER FOR QUALITY AGING
NASHVILLE, TN 372322400

SINGH, HERMAN , MD *
PRIVATE CONSULTANT
SILVER SPRING, MD 20910

SINGH, RANJIT , MBBS *
ASSOCIATE PROFESSOR
FAMILY MEDICINE
ASSOCIATE DIRECTOR, PATIENT SAFETY RESEARCH CTR
UNIVERSITY OF BUFFALO
BUFFALO, NY 14215

SNYDER, RITA , PHD, RN
PROFESSOR AND ASSOCIATE DEAN FOR RESEARCH AND EVALUATION
COEE ENDOWED CHAIR IN HEALTH INFORMATICS
QUALITY AND SAFETY EVALUATION
COLLEGE OF NURSING
UNIVERSITY OF SOUTH CAROLINA
COLUMBIA, SC 292084001

VAWDREY, DAVID KENT, PHD *
ASSISTANT PROFESSOR OF CLINICAL BIOMEDICAL INFORMATICS
COLUMBIA UNIVERSITY
DEPT. OF BIOMEDICAL INFORMATICS
NEW YORK, NY 10032

VO, NGHIA M, FACS, MD *
CONSULTANT
GENERAL SURGEON (VASCULAR)
LEESBURG, VA 20176

WEARS, ROBERT L, MD, MS
PROFESSOR
DEPARTMENT OF EMERGENCY MEDICINE
UNIVERSITY OF FLORIDA HEALTH SCIENCE CENTER
JACKSONVILLE, FL 32209

WILBUR, JOELLEN , PHD
PROFESSOR
RUSH UNIVERSITY
CHICAGO, IL 60612

ZHANG, QI , PHD *
ASSISTANT PROFESSOR
SCHOOL OF COMMUNITY AND ENVIRONMENTAL HEALTH
OLD DOMINION UNIVERSITY
NORFOLK, VA 23529

SCIENTIFIC REVIEW ADMINISTRATOR

PONCE-GONZALEZ, ILEANA M., CNC, MD, MPH
DIVISION OF SCIENTIFIC REVIEW
OFFICE OF EXTRAMURAL RESEARCH, EDUCATION
AND PRIORITY POPULATIONS
540 GAITHER ROAD, SUITE 2024
AGENCY FOR HEALTHCARE RESEARCH & QUALITY
ROCKVILLE, MD 20850

GRANTS TECHNICAL ASSISTANT

MANUEL, DIANE
DIVISION OF SCIENTIFIC REVIEW
OFFICE OF EXTRAMURAL RESEARCH, EDUCATION
AND PRIORITY POPULATIONS
AGENCY FOR HEALTHCARE RESEARCH AND QUALITY
540 GAITHER ROAD, SUITE 2000
ROCKVILLE, MD 20850

* Temporary Member. For grant applications, temporary members may participate in the entire meeting or may review only selected applications as needed.

Consultants are required to absent themselves from the room during the review of any application if their presence would constitute or appear to constitute a conflict of interest.
